# Evaluation of radiation treatment plan quality in head and neck cancer: a comparative analysis of RapidArc technique with flattening filter and flattening filter-free photon beams

**DOI:** 10.3332/ecancer.2025.1854

**Published:** 2025-02-20

**Authors:** Atul Mishra, Sumanta Manna, Kailash Kumar Mittal, Sharad Singh, Neha Yadav

**Affiliations:** 1 Department of Radiation Oncology, Uttar Pradesh University of Medical Sciences, Saifai, Etawah 206130, Uttar Pradesh, India; 2 Department of Radiation Oncology, Kalyan Singh Super Specialty Cancer Institute, C. G. City, Lucknow 226002, Uttar Pradesh, India; 3 Department of Radiation Oncology, Apollomedics Super Speciality Hospitals, Lucknow 226012, Uttar Pradesh, India; a https://orcid.org/0000-0002-3077-8628; b https://orcid.org/0000-0003-4079-1591; c https://orcid.org/0000-0001-8770-880X; d https://orcid.org/0009-0007-0607-152X; e https://orcid.org/0000-0002-7712-2415

**Keywords:** intensity modulated, linear accelerator, radiotherapy, photons, radiotherapy planning, radiotherapy, conformal, FFF, FF, RapidArc

## Abstract

**Aim:**

This study aims to evaluate the treatment plan quality for oral cavity cancers in the head and neck region using the RapidArc (RA) technique with both flattening filter (FF) and flattening filter-free (FFF) photon beams.

**Materials and methods:**

In this analytical study, treatment plans for 12 patients originally planned with a 6 MV FF photon beam were recreated using the RA technique with a 6 MV FFF photon beam. Identical beam parameters and planning objectives were maintained for both sets of plans to facilitate comparison. All plans were evaluated based on planning indices and doses to organs at risk (OAR).

**Results:**

A significant dose variation was found in the minimum (D_min_) and mean (D_mean_) doses of the high-risk planning target volume between FF and FFF photon beam RA plans. However, the dose distribution for the low-risk planning target volume was equivalent between the two techniques. The FFF-RA plans demonstrated superior conformity and homogeneity indices compared to the FF plans, with these differences being statistically significant. In addition, the FF-RA plans showed higher doses to the parotid glands, eyes and lenses than the FFF plans. The FFF plans also showed significantly shorter beam-on treatment times and a higher gamma passing index rate compared to the FF plans.

**Conclusion:**

In contrast to the FF photon beam, an FFF photon beam-oriented RA plan provides significant OAR sparing without losing the quality of the treatment plan. High monitor units and beam on time are major highlights of the RA plan with FFF beam.

## Introduction

Head and neck (H&N) cancers have a history of being linked with a late diagnosis and poor prognosis [1]. A significant number of patients have locally aggressive disease that requires multimodality treatment. Oral cavity tumours are a common type of H&N cancer.

Oral cavity tumours are a prevalent type of H&N cancer, accounting for approximately 70% of all reported cases in this category [2]. Radiotherapy remains a crucial component in the treatment of these tumours and plays a vital role in many therapeutic approaches for oral cavity cancer [3].

However, it is incredibly challenging to enhance the local dose for H&N cancer using traditional radiation technology (i.e., parallel-opposed fields with lower anterior neck irradiation for phase 1 and parallel-opposed fields for phase 2); as a result, the 5-year survival rate for H&N cancer remains between 30% and 50%, depending on the stage and location of the tumour [4–7].

The capacity to develop intensity-modulated beams with a multi-leaf collimator and inverse planning enables the use of flattening filter-free (FFF) beams to achieve a more conformal distribution, regardless of beam shape, making the flattening filter (FF) less significant. Furthermore, as Xiao *et al* [8] pointed out, neither patients nor targets are flat; therefore, FFF fields may be advantageous for moderate or even large targets. The treatment of H&N cancers presents unique challenges because it often affects several critical organs. RapidArc (RA) and intensity-modulated radiation therapy (IMRT) are commonly used treatment methods for these types of cancers because they offer better radiation dose control and help protect nearby critical organs, ultimately leading to improved survival rates and a better quality of life for patients [9]. Numerous studies have explored the practical use of FFF beams, focusing on understanding the properties of FFF beams and assessing their suitability in treatment planning and delivery [10-13]. Radiotherapy machines in contemporary medicine are equipped with FFF beams. Employing FFF beams in plans for treatments such as volumetric modulated arc therapy (VMAT) and IMRT offers numerous potential benefits. These advantages encompass heightened dose delivery rates, diminished collimator scatter, decreased leakage from the machine head and lowered radiation exposure to areas outside the intended treatment field for the patient [14–20]. Cashmore *et al* [20] documented instances of radiation leakage and reduced peripheral dose when utilising FFF beams in treatment plans targeting the thyroid, lungs, ovaries and testis [21, 22]. However, there have been limited studies conducted on the use of FFF beams for treating H&N cancers, which is insufficient to establish reliable statistical data. Some studies involving larger treatment targets have shown that FFF plans may have less uniformity. In addition, these studies have indicated that FFF beams can yield treatment plan quality and dosimetric parameters similar to FF beams, with no observed improvement in sparing critical organs [21, 22].

To keep up with the evolving sophistication of treatments, quality assurance (QA) for RA has advanced significantly. Various commercial 2D and 3D ionisation chambers or diode detector arrays have recently become popular, enabling quick results to verify absolute dose. Detector arrays are progressively replacing traditional techniques like film dosimetry and point dose measurements in an ionisation chamber. These tools have allowed centres to improve their QA and treat more patients with RA [23]. Moreover, the resolution of detector arrays is restricted [24]. For patient-specific QA, the widely used gamma (γ) passing criteria is a 3 mm distance to agreement (DTA) with a dose difference of 3% and the deviation should be less than 5% in such setting; treatment plans may be delivered without any correction [25, 26].

The current study aims to evaluate the treatment plan quality for oral cavity cancers in the H&N region using the RA technique with both FF and FFF photon beams.

## Materials and methods

Twelve patients with H&N cancer who underwent radical resection and required post-operative radiotherapy for oral tongue (anterior tongue) squamous cell carcinomas were selected for this study. Nine males and three females were taken in the current study. The disease was staged as per the American Joint Committee on Cancer Staging Guidelines 8th edition (2017), and stagewise distribution was: Stage II - Three patients, Stage III - Seven patients and Stage IV - Two patients. All twelve patients were simulated with a computed tomography (CT) simulator in the supine position. The CT simulations were performed with a helical scanner with a 3.0 mm slice thickness. The tumour volumes, gross tumour volume, clinical target volume (CTV), planning target volume (PTV) and contouring of organs at risk (OARs) were done according to RTOG protocol [27]. Two sets of RA with dual-arc plans were performed with jaw tracking using 6-MV FF and FFF beams. All RA plans were generated using the Eclipse treatment planning system (v15.6; Varian Medical Systems, Palo Alto, CA, USA). A photon optimiser (PO; Version15.6.06, Varian Medical Systems) was selected for inverse optimisation based on physical and biological objectives with a 2.5 mm dose grid resolution and Anisotropic Analytical Algorithm (AAA). All plans were generated for PTV high risk (PTV_HR) 60Gy/30# and PTV low risk (PTV_LR) 54Gy/30#, a uniform 5 mm margin was taken around the CTV to create the PTV in all cases. Dose constraints given for OAR planning were Parotid Gland (Both) mean dose <25 Gy, Brainstem D_max_ <54 Gy, Eyes (globe) Mean <35 Gy and D_max_ <54 Gy and Lens D_max_ <7 Gy. The spinal cord (SC) dose limit (D_max_) was kept at <45 Gy. A Varian TrueBeam accelerator equipped with 120 leaves Millennium multi-leaf collimator (M120, MLC) was used to develop all RA plans with a maximum dose rate of 600 monitor units (MU)/min and 1,400 MU/min for the FF and FFF photon beams, respectively. Patient plan optimisation and sequencing parameters were kept as per standard protocol.

### Plan assessment and validation

All RadpidArc plans (with FF and FFF) were evaluated for HI, CI, D_max_, D_mean_, D_98_, D_50_, D_2_, V_95_, V_107_ and dose to OAR using dose volume histogram (DVH). Gradient index (GI) is an indicator of dose fall-off. It assesses the dose of radiation gradient beyond the target. GI was defined as a ratio of V_95_% prescription isodose dose (PID) over V_50_% PID [28, 29]. Low GI and High Gradient Index were defined as the ratio of V_25_% PID over V_50_% PID and V_50_% PID over V_90_% PID, respectively, where V_25_%, V_50_% andV_90_% were volumes receiving 25%, 50% and 90% of the PID, respectively [28, 29]. 

The doses to the OARs were recorded for all 12 patients, with mean ± standard deviation (SD) values for each patient's plans. The doses calculated by the treatment planning system (TPS) were compared with measured doses using patient-specific QA. In this study, patient-specific QA for RA used a 3 mm DTA and 3% dose difference criteria, with a threshold value set at 5% [25]. Therefore, both FF and FFF RA plans were irradiated and compared using an electronic portal imaging detector (EPID). Furthermore, the dose difference analysis was defined as the TPS-calculated dose at a specific point minus the measured dose at the same point, divided by the measured dose at that point (Equation 1) [26].

PV= {[TPSPD (FF or FFF) −Measured Dose (EPID)]/ Measured Dose (EPID)} ×100 (1)

Where PV defines percentage variation; TPSPD, TPS Planned Dose; FF, flattened filter; FFF, FFF and EPID, Electronic Portal Imaging Detector for equation 1.

### Statistical analysis

The mean and SD were calculated for continuous variables to summarise the central tendency and dispersion of the data. Comparisons between the Rapid with FF and RA with FFF groups were performed using two-tailed paired *t*-tests. Statistical analyses were conducted using R programming software. A *p*-value of less than 0.05 was considered statistically significant.

## Results

**Figure 1.**
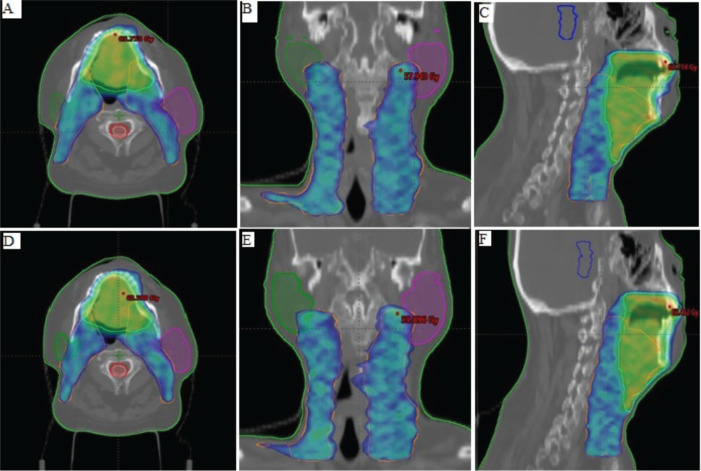
Displays the dose colourwash distribution covering the high-risk (cyan) and low-risk PTV (orange). In the figure, A–C represent plan distributions with FF beams in the three planes (axial, coronal and sagittal), while D–F represent plan distributions with FFF beams.

In the current study, PTV_HR and PTV_LR mean volumes were 265.12 ± 64.65 and 385.41 ± 112.84 cc, respectively. The average PTV coverage for all patients in both FF and FFF RA techniques could achieve 95% of the prescription dose (60 and 54 Gy) to 95% of the PTV and didn’t exceed 109%. Figure 1 shows the dose colour wash distribution for FF and FFF beam plans.

Figure 1 displays the dose colour wash distribution covering the high-risk (cyan) and low-risk PTV (orange). In the figure, A–C represent plan distributions with FF beams in the three planes (axial, coronal and sagittal), while D–F represent plan distributions with FFF beams.

The D_98_, D_95_, D_2_, D_50_, D_mean_, D_max_, D_min,_ V_95_ and V_107_ were recorded for PTV_HR and PTV_LR (Table 1). All these values were found statistically not significant except D_min,_ V_107_ and D_mean_ for PTV_HR of FF and FFF plans. The average HI values were found to be 0.07 ± 0.01 and 0.08 ± 0.01 for FF and FFF of PTV_HR, respectively. The average HI values were found to be 0.08 ± 0.01 and 0.09 ± 0.01 for FF and FFF of PTV_LR, respectively. HI values for both PTVs were found statistically significant (*p* = 0.02 for PTV_HR and *p* = 0.00 for PTV_LR). The average CI values were found similar for both PTVs for FF and FFF, respectively, which is statistically significant (*p* = 0.03). Table 2 presents the dosimetric parameters for normal tissue and OARs for all patients.

**Figure 2.**
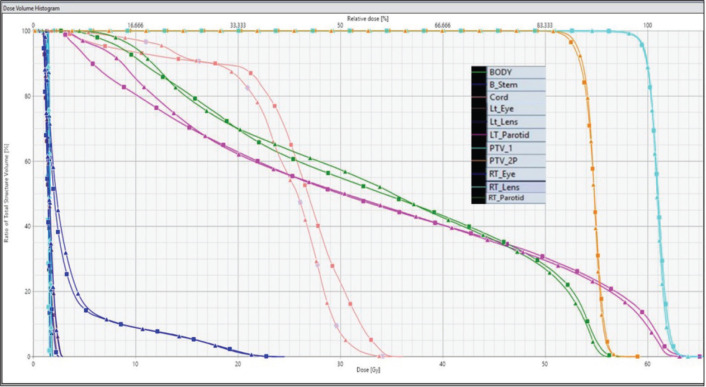
DVHs for PTVs and OARs. Lines with triangles represent FF data, and those with squares represent FFF data.

Statistically significant differences were observed in normal tissue parameters such as V_90_, V_50_ and low dose gradient index (LGI). However, parameters such as V_95_, V_25_, GI and high dose gradient index (HGI) showed no statistically significant differences between both techniques. Figure 2 illustrates the DVHs for PTVs and OARs of FF and FFF beam plans.

**Figure 3.**
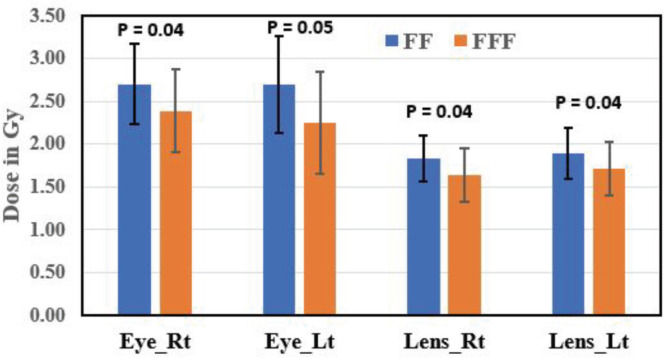
A bar chart comparing the dose (Mean ± SD) in Gy to the OAR, for the right eye (Eye_Rt), left eye (Eye_Lt), right lens (Lens_Rt) and left lens (Lens_Lt), in both FF and FFF plans.

**Figure 4.**
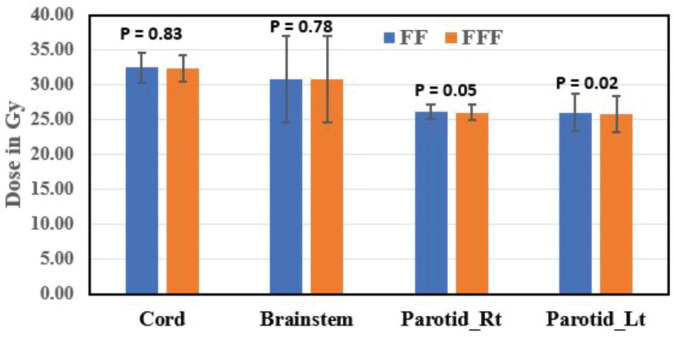
A bar chart comparing the dose (Mean ± SD) in Gy to the OAR, cord, brainstem, parotid right (Parotid_Rt) and parotid left (Parotid_Lt), in both FF and FFF plans.

The mean dose to the right parotid gland was significantly higher with FF RA plans (26.14 ± 1.08 Gy) compared to FFF RA plans (26.02 ± 1.02 Gy) (*p* = 0.05). Similarly, the mean dose to the left parotid gland was significantly higher with FF RA plans (26.04 ± 2.65 Gy) compared to FFF RA plans (25.84 ± 2.60 Gy) (*p* = 0.02). FFF plans showed a significant reduction in the right eye and left eye dose than FF plans and were found to be statistically significant (*p* = 0.04 for the right eye and *p* = 0.05 for left eye). Eye lenses (both right and left) were shown less for FFF techniques and statistically significant (*p* = 0.04). SC max dose was within the tolerance limit for FF and FFF techniques (*p* = 0.83). Statistical variations of OAR dose are represented in Figures 3 and 4.

Brainstem max dose was reported for all 12 patients and was 30.82 ± 6.17 and 30.79 ± 6.18 Gy for FF and FFF RA, respectively, and statistically not significant (*p* = 0.78).

### Gamma passing criterion

All the plans for the 12 patients were verified using EPID (a-Si 1200 portal imager).

The results showed that the gamma passing rate (the passing criteria were 3% dose difference at 3 mm DTA) of the SD in the different plans was 97% or higher.

**Figure 5.**
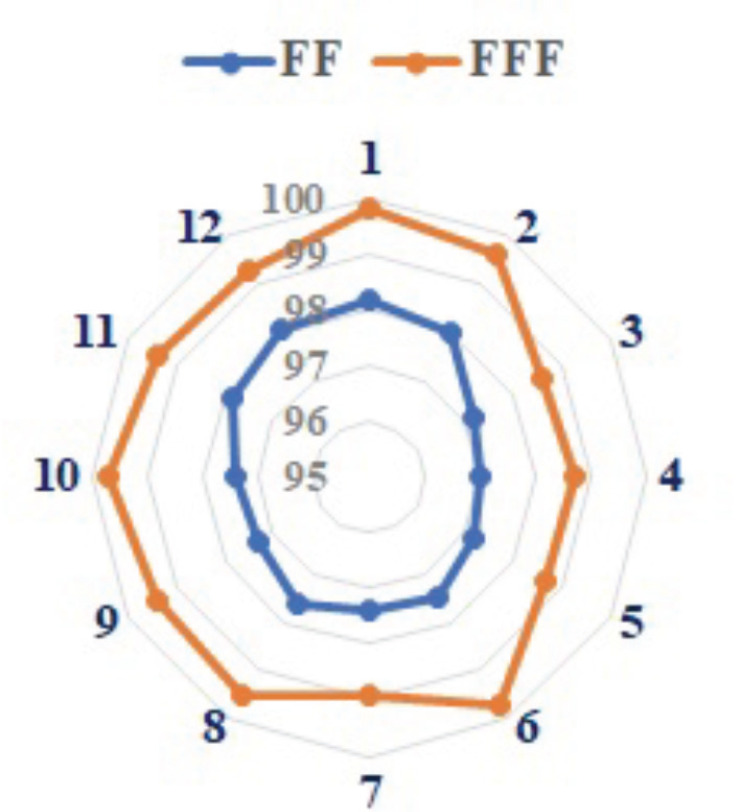
Variation of percentage of gamma passing rate for FF and FFF beam with AAA dose.

The gamma passing rate for FFF increased by 1.76% compared to FF, i.e., 99.28% versus 97.56%, respectively. Figure 5 represents the gamma index passing values for different patients.

### MU and treatment time (TT)

For the two techniques in this study, the variations in cumulative MUs were statistically significant (*p* = 0.00). FFF RA (697.18 MU) was increased by 6.41% relative to FF RA (655.22 MU) on the mean for a variation.

The MU of the FF RA plan in the H&N region was noticeably lower compared to the FFF RA plan, as shown in Table 3 (*p* = 0.00).

**Figure 6.**
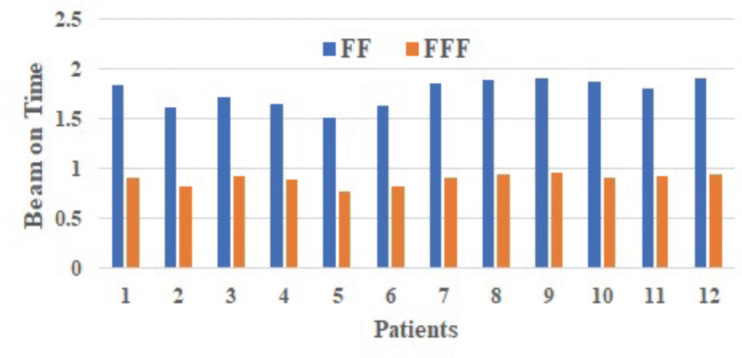
Total BOT of FF and FFF for different patients computation algorithm.

The difference between the two plans' beam on time (BOT) (minutes) was statically significant, with the FFF plan (0.90 minutes) being, on average, 49.15% faster than the FF plan (1.77 minutes) (*p* = 0.00; Table 3). The total beam-on time of FF and FFF for different patients as shown in Figure 6.

## Discussion

The current study evaluates the radiation treatment plan quality of the H&N for FF and FFF radiotherapy plans based on different dosimetric parameters and plan validation with patient-specific QA using EPID. 

Presently, the FFF RA technique has been used to treat the H&N, the lungs and a number of other regions. When compared to other radiation therapy approaches, the FFF RA technique may help reduce TT while also achieving better radiobiological impacts [21, 22]. Furthermore, there has been very little evidence of such a study being employed on patients with H&N cancer. It remains a significant challenge to improve target prescribed dose coverage, dosage homogeneity and conformity and reduce dose to OARs in H&N malignancies.

In the present study, authors found that FF and FFF RA techniques were able to achieve greater than 95% of the prescription dose (60 and 54 Gy) to 95% of the PTV and less than 109%. Kumar *et al* [2] reported similar outcomes utilising the FF and FFF RA techniques for H&N patients. Saroj *et al* [30] reported HI for FFF plans was better than FF plans and CI was clinically insignificant. In the current study, FFF plans show better HI, which is statistically significant (*p* = 0.02), and FF RA plans show better CI than FFF RA plans, which is statistically significant (*p* = 0.03). Manna *et al* [31] conducted a study on brain neoplasms planned with 6 MV FFF and VMAT and found superior conformity of the dose to the PTV, as well as a reduction in the dose to the eyes and optic nerve. In addition, there was a significant reduction in low-dose volumes and integral doses. In a similar study, Manna *et al* [32] demonstrated that for cervical cancer treatment, the use of the FFF beam and RA technique resulted in a statistically significant improvement in the conformity index, with an observed difference of 3.06%. FFF beams are clinically advantageous in the treatment of lung cancer as well as in radiation therapy for synchronous bilateral breast carcinoma [33, 34].

Kumar *et al* [29] reported that parotid, eye and lens doses were similar and statistically insignificant for H&N RA FF and FFF plans. However, in the present study shows a lesser dose in parotid (Parotid_Rt: *p* = 0.05, Parotid_Lt: *p* = 0.02), eye (Eye_Rt: *p* = 0.04, Eye_Lt: *p* = 0.05) and lens (Lens_Rt: *p* = 0.04, Lens_Lt: *p* = 0.04) for FFF RA plans and statistically significant. Kumar *et al* [29] reported that GI for FF and FFF plans were 3.85 and 3.87, respectively, and statistically insignificant (*p* = 0.96). In the present study, a similar trend was observed for GI and less LGI for FFF over FF plans, statistically significant (*p* = 0.96). However, HGI was statistically insignificant for FF and FFF RA plans (*p* = 0.16).

Saroj *et al* [35] compared the quality of IMRT treatment plans for cancer of the esophageal with and without FF photon beams. The IMRT plan with FFF beam offers significant benefits, including high MUs and less BOT. In this investigation, the MUs exhibited a statistically significant difference (*p* = 0.00). The quantity of MUs for the RA FF plan (655.22 ± 72.43 MU) was found to be lesser compared to the RA FFF plan (697.18 ± 65.68 MU). The BOT between the two plans was also statistically significant. Specifically, RA FFF plans demonstrated a shorter BOT (0.90 ± 0.06 minutes) compared to the longer BOT (1.77 ± 0.13 minutes) for RA FF plans (*p* = 0.00). The observed increase in MUs for FFF-RA plans, as opposed to FF-RA plans, might be attributed to the necessity for increased modulation when utilising FFF beams to ensure uniform dose distribution in larger tumours, resulting in a higher MU count. Nevertheless, the higher dose rate associated with FFF beams contributes to an overall reduction in beam delivery time. Reducing treatment delivery time would be beneficial for patients susceptible to motion.

As per recommendations, SC and brainstem dose (45 and 54 Gy, respectively) were both efficacious and met the specified tolerance criteria [29, 36]. In the current study, the authors stated that the mean dose to the two essential organs (SC and Brainstem) for RA FFF plans accomplished the above criteria and slightly decreased dose as compared to RA FF plans (*p* = 0.83 and *p* = 0.78, respectively).

AAPM task group 119 suggests an appropriate threshold indicated as a percentage of points meeting gamma criteria of 3%/3 mm: 94.2% for individual field assessments and 92.4% for combined irradiations assessed using radiographic film [25, 37–39]. Ji *et al* [40] evaluated the feasibility of using FFF beams for whole-brain radiotherapy while sparing the hippocampus. They assessed target and organ-at-risk parameters for both FF and FFF beams and concluded that the differences in the gamma index were negligible. In the current study, we found that the mean gamma index passing criteria was greater than 97% at 3%/3 mm for both techniques, which shows good results of our plans. FFF was superior to FF with a betterment of 1.76% (99.28% versus 97.56%). 

The limitations of the study included a small number of participants and an inequitable distribution of male as well as female patients. It is therefore recommended that a newer study be conducted with a larger number of participants for high validity and that long-term clinical follow-up may also be required to determine whatever radiotherapy toxicities. Subsequent studies could explore potential radiobiological effects and further investigate the FFF technique to minimise TT. The rapid advancement in the radiation treatment of mid and advanced H&N cancers suggests promising avenues for future research.

## Conclusion

From the present study, it can be concluded that FFF-based RA plans were better than FF plans in the context of target coverage, OAR constraints, HI and CI. Moreover, FFF RA plans to prove to be effective in reducing intrafraction errors, particularly in H&N tumour cases, as they exhibit significantly shorter TT compared to FF plans.

## Conflicts of interest

The authors state that they have no conflicting interests in this manuscript.

## Funding

No funding was received for this work.

## Ethical approval

Since this is a retrospective study involving previously treated patients, ethical approval was exempted by the institute.

## Figures and Tables

**Table 1.** Dosimetric and plan quality indices for the PTV using 6 MV FF and 6 MV FFF RA plans.

*N* = 12	Parameters	6 MV_FF	6 MV_FFF	*p*-value
		Mean ± SD		
PTV_HR	Volume (cc)	265.12 ± 64.65		
	D_max_ (Gy)	65.20 ± 0.79	65.41 ± 0.69	0.20
	D_min_ (Gy)	52.79 ± 1.84	51.69 ± 1.32	**0.00**
	D_mean_ (Gy)	60.94 ± 0.12	61.02± 0.11	**0.03**
	D_98%_ (Gy)	58.62 ± 0.29	58.43 ± 0.39	0.07
	D_95%_ (Gy)	59.8 ± 0.09	59.79 ± 0.13	0.06
	V_95%_ (cc)	264.44 ± 64.33	263.79 ± 63.95	0.14
	V_107%_(cc)	0.14 ±0.17	0.46 ± 0.63	**0.02**
	CI = (TV_PIV_)^2^/ (TV × PIV)	0.84 ± 0.03	0.83 ± 0.03	**0.03**
	HI= D_2_-D_98_/D_50_	0.07 ± 0.01	0.08 ± 0.01	**0.02**
PTV_LR	Volume (cc)	385.41 ± 112.84		
	D_max_ (Gy)	59.34 ± 0.44	59.45± 0.34	0.46
	D_min_ (Gy)	47.05 ± 1.00	46.53 ± 0.99	0.16
	D_mean_ (Gy)	54.81 ± 0.17	54.82 ± 0.17	0.67
	D_98%_ (Gy)	52.30 ± 0.35	52.19 ± 0.38	0.06
	D_95%_ (Gy)	53.00 ± 0.31	52.92 ± 0.29	0.06
	V_95%_ (cc)	383.31 ±111.82	382.89 ± 111.91	0.08
	CI = (TV_PIV_)^2^/ (TV × PIV)	0.84 ± 0.03	0.83 ± 0.03	**0.03**
	HI=D_2_-D_98_/D_50_	0.08 ± 0.01	0.09 ± 0.01	**0.00**

FF: flattening filter plan, FFF: flattening filter-free, Da (Gy): Dose (Gy) absorbed dose by particular % or volume cm^3^, Va: Percentage of organ volume for particular dose, HI: Homogeneity Index, CI: Conformity Index, SD: Standard Deviation, PTV: Planning Target Volume, HR: High Risk, LR: Low Risk, Bold *p*-values: statistical significance (*p* < 0.05).

**Table 2.** Dosimetric parameters for normal tissue and OARs.

*N* = 12	Parameters	6 MV_FF	6 MV_FFF	*p*-value
		Mean ± SD		
Normal tissue	V_95_ (cc)	314.75 ± 73.93	315.19 ± 72.99	0.58
	V_90_ (cc)	690.63 ± 82.11	682.82 ± 79.80	**0.01**
	V_50_ (cc)	1,559.54 ±198.27	1,591.12 ± 213.61	**0.00**
	V_25_ (cc)	2,898.70 ± 434.54	2,906.60 ± 456.57	0.60
	GI = V_95_ / V_50_	0.21 ±0.06	0.22 ±0.09	0.45
	LGI = V_25_ / V_50_	1.86 ±0.10	1.82 ± 0.10	**0.00**
	HGI = V_50_/ V_90_	2.26 ± 0.16	2.48 ± 0.49	0.16
OARs	SC (D_max_)	32.47 ± 2.18	32.37 ± 1.88	0.83
	Brainstem (D_max_)	30.82 ± 6.17	30.79 ± 6.18	0.78
	Parotid_Rt (D_mean)_	26.14 ± 1.08	26.02 ± 1.09	**0.05**
	Parotid_Lt (D_mean)_	26.04 ± 2.65	25.84 ± 2.60	**0.02**
	Eye_Rt (D_max_)	2.70 ± 0.47	2.39 ± 0.48	**0.04**
	Eye_Lt (D_max_)	2.69 ± 0.56	2.25 ± 0.60	**0.05**
	Lens_Rt (D_max_)	1.83 ± 0.27	1.64 ± 0.31	**0.04**
	Lens_Lt (D_max_)	1.89 ± 0.30	1.71 ± 0.32	**0.04**

FF: flattening filter plan, FFF: flattening filter-free plan, Va: Percentage of organ volume for particular dose, GI: Gradient Index, LGI: Low Gradient Index, HGI: High Gradient Index, Rt: Right, Lt: Left, OAR: Organ at risk, SD: Standard Deviation, Bold *p*-values: statistical significance (*p* < 0.05).

**Table 3.** Comparison of MU, BOT and gamma passing rate (GP) for 6 MV FF and 6 MV FFF RA plans.

Index	6 MV_FF	6 MV_FFF	p-value
	Mean ± SD		
MU	655.22 ± 72.43	697.18 ± 65.68	0.00
BoT	1.77 ± 0.13	0.90 ± 0.06	0.00
GP	97.56 ± 0.37	99.28 ± 0.42	0.00

FF: flattening filter plan, FFF: flattening filter-free plan, MU: Monitor Units, BoT: Beam on Time, GP: Gamma Passing Rate
